# Black Raspberries Suppress Colorectal Cancer by Enhancing Smad4 Expression in Colonic Epithelium and Natural Killer Cells

**DOI:** 10.3389/fimmu.2020.570683

**Published:** 2020-12-14

**Authors:** Yi-Wen Huang, Chien-Wei Lin, Pan Pan, Tianjiao Shan, Carla Elena Echeveste, Yue Yang Mo, Hsin-Tzu Wang, Mohammed Aldakkak, Susan Tsai, Kiyoko Oshima, Martha Yearsley, Jianbo Xiao, Hui Cao, Chongde Sun, Ming Du, Weibin Bai, Jianhua Yu, Li-Shu Wang

**Affiliations:** ^1^ Department of Obstetrics & Gynecology, Medical College of Wisconsin, Milwaukee, WI, United States; ^2^ Division of Biostatistics, Medical College of Wisconsin, Milwaukee, WI, United States; ^3^ Division of Hematology and Oncology, Department of Medicine, Medical College of Wisconsin, Milwaukee, WI, United States; ^4^ Department of Surgery, Medical College of Wisconsin, Milwaukee, WI, United States; ^5^ Department of Pathology, Johns Hopkins University, Baltimore, MD, United States; ^6^ Department of Pathology, The Ohio State University, Columbus, OH, United States; ^7^ Institute of Chinese Medical Sciences, State Key Laboratory of Quality Research in Chinese Medicine, University of Macau, Macau, Macau; ^8^ Laboratory of Fruit Quality Biology/Zhejiang Provincial Key Laboratory of Horticultural Plant Integrative Biology/The State Agriculture Ministry Laboratory of Horticultural Plant Growth, Development and Quality Improvement, Zhejiang University, Zijingang Campus, Hangzhou, China; ^9^ School of Food Science and Technology, National Engineering Research Center of Seafood, Dalian Polytechnic University, Dalian, China; ^10^ Department of Food Science and Engineering, Institute of Food Safety and Nutrition, Guangdong Engineering Technology Center of Food Safety Molecular Rapid Detection, Jinan University, Guangzhou, China; ^11^ Department of Hematology and Hematopoietic Cell Transplantation, Comprehensive Cancer Center, City of Hope National Medical Center, Duarte, CA, United States

**Keywords:** black raspberries, Smad4, natural killer cells, colorectal (colon) cancer, human clinical trials

## Abstract

Innate immune cells in the tumor microenvironment have been proposed to control the transition from benign to malignant stages. In many cancers, increased infiltration of natural killer (NK) cells associates with good prognosis. Although the mechanisms that enable NK cells to restrain colorectal cancer (CRC) are unclear, the current study suggests the involvement of Smad4. We found suppressed Smad4 expression in circulating NK cells of untreated metastatic CRC patients. Moreover, NK cell-specific Smad4 deletion promoted colon adenomas in DSS-treated *Apc^Min/+^* mice and adenocarcinomas in AOM/DSS-treated mice. Other studies have shown that Smad4 loss or weak expression in colonic epithelium associates with poor survival in CRC patients. Therefore, targeting Smad4 in both colonic epithelium and NK cells could provide an excellent opportunity to manage CRC. Toward this end, we showed that dietary intervention with black raspberries (BRBs) increased Smad4 expression in colonic epithelium in patients with FAP or CRC and in the two CRC mouse models. Also, benzoate metabolites of BRBs, such as hippurate, upregulated Smad4 and Gzmb expression that might enhance the cytotoxicity of primary human NK cells. Of note, increased levels of hippurate is a metabolomic marker of a healthy gut microbiota in humans, and hippurate also has antitumor effects. In conclusion, our study suggests a new mechanism for the action of benzoate metabolites derived from plant-based foods. This mechanism could be exploited clinically to upregulate Smad4 in colonic epithelium and NK cells, thereby delaying CRC progression.

## Introduction

Understanding the progression of benign adenoma and high-grade dysplasia to colorectal cancer (CRC) is critical for developing therapeutics that prevent or treat the disease. As reported for a variety of cancers, a greater number of tumor-infiltrating natural killer (NK) cells associates with good prognosis of CRC (1, 2). NK cells, a subset of innate lymphoid cells (3), are the first line of defense against tumor cells and viruses (4). They can directly lyse tumor cells, including those from adenocarcinoma and its precursors, without prior activation (3). In patients with acute myeloid leukemia (AML), killer immunoglobulin-like receptor-mismatched donor NK cells were shown to efficiently eradicate AML cells, enhance patient survival, and prevent relapse in the haplotype-mismatched transplant setting (5). NK cell immunosuppression in CRC includes phenomena such as low NKG2D ligands (6), low perforin/granzymes (7), low IFN-gamma production (8), low degranulation capabilities (8), a decrease of CD16+CD56+ NK cells (9), and NK cell polarization toward a pro-inflammatory (10) and pro-angiogenic (11, 12) phenotype. However, targeting NK cells in CRC is only just emerging as a treatment strategy. In 2018, the first clinical trial reported that a combination of adoptive transfer of expanded NK cells combined with standard chemotherapy had anti-tumor potential in advanced CRC patients (13).

Smad4, is a known tumor suppressor in human CRC in epithelium (14, 15). However, its role in innate immune cells is unclear, though loss or weak expression of Smad4 is known to associate with poor survival in CRC patients (14, 15). Furthermore, Smad4 loss is also seen in adenomas of patients with familial adenomatous polyposis (FAP) (16), suggesting that it contributes to the progression of CRC in populations at high risk for that disease. Why Smad4 is downregulated during cancer is unclear, but it is not likely due to mutation, as only 8.6% of cases in a cohort of 744 primary sporadic CRC patients had Smad4 mutations (17). Other factors that regulate Smad4 expression are not well understood, though Smad4 drives the development of activated T cells that participate in immune surveillance to protect the host from cancers, including CRC (18). Interestingly, Smad4 was shown to promote TGF-beta-independent NK cell homeostasis and maturation and antitumor immunity in a murine metastatic melanoma model (19, 20). However, the role of Smad4 in NK cells during CRC progression remains to be elucidated.

Metabolites derived from plant-based foods can boost the anti-CRC functions of NK cells (21). Thus, several lines of evidence suggest that nutrients or phytochemicals can modulate NK cell function (21–23), though exploration of mechanisms is lagging. However, inositol derived from food can modulate PI3K signaling in NK cells (24). In addition, food-derived nutrients and bioactive components are metabolized in the gut, where they are transformed into compounds whose biological activities differ from those of the parental compounds (25–33).

Freeze-dried black raspberries (BRBs) at 5%–10% of the diet have been reported to suppress tumors in multiple organ sites in animal models and humans (34–39). Our previous study showed that depleting NK cells significantly promoted CRC development (40). Moreover, BRBs significantly suppressed CRC progression and increased the number of tissue-infiltrating NK cells in *Apc* ^Min/+^ mice treated overnight with DSS, in mice treated with AOM, and in DSS models (40). BRB intervention also enhanced the number and functionality of NK cells in CRC patients (40). In the current study, we determined how Smad4 affects CRC progression and whether dietary BRBs increase Smad4 expression in mouse models of CRC. We also determined whether BRBs and their metabolites modulate Smad4 expression in human CRC and FAP and in human NK cells. Our results suggest that Smad4 could play tumor suppressor roles in both colonic epithelium and NK cells as CRC progresses, thus representing a novel molecular therapeutic target for preventing and/or treating this disease. Also, metabolites. These new mechanisms might suggest that use of those metabolites to upregulate Smad4 in both colonic epithelium and NK cells could delay the progression of CRC.

## Materials and Methods

### Human Specimens

**Figure 1:**
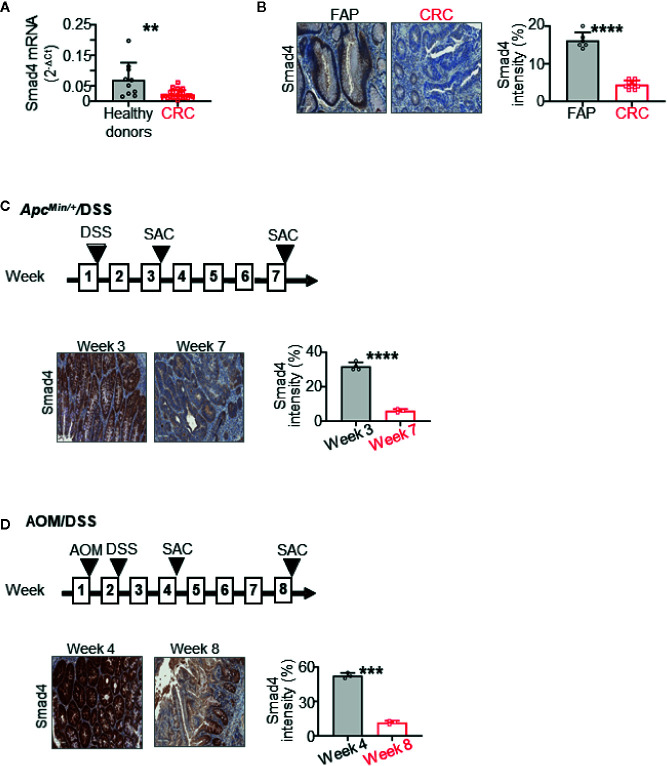
Smad4 expression is decreased in human colorectal cancer (CRC) and during the CRC progression in *Apc^Min/+^* mice treated overnight with dextran sulfate sodium (DSS). It also decreases during the progression of CRC in wild-type mice treated with azoxymethane (AOM) and then overnight with DSS. **(A)** Smad4 mRNA expression in circulating NK cells from healthy donors (n=10) and untreated metastatic CRC patients (n=24) by RT-qPCR. **(B)** Representative immunohistochemistry (IHC) staining of Smad4 in colorectal tissues from familial adenomatous polyposis (FAP) (n=6) and CRC (n=9) patients. Quantification of Smad4 staining intensity. **(C)** Representative IHC images and quantitative staining of Smad4 on colon adenoma in the *Apc^Min/+^*/DSS mouse model (n=3–4 for each time points). **(D)** Representative IHC images and quantitative staining of Smad4 on colon adenocarcinoma in the AOM/DSS mouse model (n=3 for each time points). SAC: mouse euthanized. The circles and squares on the bar graphs denote data from individual specimens. **P < 0.01; ***p < 0.001; ****P < 0.0001.

For **Figure 1A**, frozen PBMC samples from healthy human subjects (n=10, 1x10^6^ cells per patient) were obtained from Cooperative Human Tissue Network and those from colon cancer patients (n=24, 1x10^6^ cells per patient) were collected from the Tissue Bank at Department of Surgery, Medical College of Wisconsin. For **Figure 1B**, immunohistochemical staining of paraffined-embedded colorectal samples from CRC patients (n=9) and FAP patients (n=6) were collected from Cooperative Human Tissue Network. A table listing the available clinical information (age and gender) was provided in **Supplementary Table 1**. For **Figures 3E, F**, about 40 ml of blood from each healthy human individual (n=5) was obtained from the blood bank at Wisconsin Diagnostic Laboratory from American Red Cross, Milwaukee, Wisconsin and no clinical information is available because of the intent of protecting the privacy of blood donors.

For **Figures 3C, D**, procurement of human blood from CRC and FAP patients in black raspberry intervention clinical trials was approved by the institutional review boards of The Ohio State University, University of Texas at San Antonio, Cleveland Clinics and Medical College of Wisconsin, as described in details in our previous publications (41, 42). All the patients enrolled in our study provided written informed consent. Colon specimens were histopathologically confirmed and paraffin-embedded tissues were used for molecular studies.

### Human NK Cell Isolation and Treatments

Human NK cells were isolated from fresh peripheral blood from healthy subjects and PBMC from healthy subjects and colon cancer patients using the NK Cell Isolation Kit (Miltenyi Biotec, Auburn, CA, USA) according to the manufacturer’s protocol but with minor modifications, as described previously (43). For **Figures 3E, F**, freshly isolated NK cells from peripheral blood from healthy subjects were cultured in RPMI1640+10% FBS+1% P/S and immediately treated with hippurate, 3-hydroxyphenyl-acetic acid, 2,4,6-trihydroxybenzoic acid monohydrate at 100 nM and 1 µM, or DMSO (Millipore Sigma, St. Louis, MO, USA) for 16 h. The cells were collected for real-time PCR and western blot as stated below.

### Animal Bioassay

All animal protocols followed the institutional guidelines for animal care and were approved by the Medical College of Wisconsin Animal Care and Use Committee as stated previously (40). The *Apc^Min^* ^/+^ and *Smad4* ^fl/fl^ mice were purchased from the Jackson Laboratory (Bar Harbor, ME). The NK*^p46^* ^-iCre^ mice were provided by Dr. Eric Vivier (Centre d’Immunologie de Marseille-Luminy, Marseille, France). All the mice were on a C57BL/6 background. The *Smad4* ^ΔNK^ mice were bred by crossing *Smad4* ^fl/fl^ and NK*^p46^* ^-iCre^ mice to delete both *Smad4* alleles in NK cells.

The American Institute of Nutrition (AIN)-76A diet was purchased from Dyets (Bethlehem, PA), and BRB powder came from Berri Products (Corvallis, OR). Azoxymethane (AOM) was obtained from Millipore Sigma and dextran sulfate sodium (DSS, 36,000–50,000 M.W.) from MP Biochemicals (Santa Ana, CA).

Four-to-five-week-old *Apc^Min^* ^/+^ and *Smad4* ^ΔNK^ *Apc* ^Min/+^ mice were given 5% DSS in their drinking water overnight. Five-to six-week-old WT and *Smad4* ^ΔNK^ mice received an i.p. injection of AOM (15 mg/kg, body weight). One week after the injection, the mice received 5% DSS in their drinking water overnight. Two and six weeks after the DSS treatment, all the mice were euthanized by CO_2_ asphyxiation. Their colons were fixed in formalin and embedded in paraffin (FFPE), and hematoxylin and eosin (H&E) staining was performed. Certified pathologists examined the entire colon under high-power magnification (20×) in a blinded manner to determine the numbers and sizes of the colonic polyps.

### Immunohistochemistry

FFPE rectal and colon tissue blocks were cut into 4 μm sections, and immunohistochemistry (IHC) was conducted as previously described (40–42). A Dako Autostainer (Santa Clara, CA, USA) was used to stain the slides with a primary antibody to Smad4 (1:50, ab40759) that we obtained from Abcam (Cambridge, MA, USA). Stained slides were photographed at 20× magnification, and only staining in the adenoma and adenocarcinoma areas was quantified, as previously described (40–42).

### RT-qPCR

Hippurate, 3-hydroxyphenyl-acetic acid, and 2,4,6-trihydroxybenzoic acid monohydrate treated NK cells (2x10^5^ cells per dose, duplicate) were isolated to determine Smad4 gene expression, Total mRNA was reverse-transcribed using iScript RT Supermix (Bio-Rad, Hercules, CA, USA). PCR was performed as described previously (40–42) with iTaq Univer SYBR Green Supermix (Bio-Rad). Human Smad4 primers were purchased from Integrated DNA Technologies (IDT), Inc. (Coralville, IA, USA). Relative expression of a gene in cells was determined by comparing the threshold cycle (Ct) of the gene against the Ct of a housekeeping gene, Gapdh.

### Western Blotting

Western blot analysis was performed as described previously (19, 43). Anti-GZMB antibody (catalog 4275) was purchased from Cell Signaling Technology (Danvers, MA, USA). Briefly, 15μg of protein lysate from hippurate, 3-hydroxyphenyl-acetic acid, and 2,4,6-trihydroxybenzoic acid monohydrate treated NK cells (1x10^6^ cells per dose, duplicate) was loaded onto 10% Mini-PROTEAN TGX Gel (Bio-Rad), and transferred to polyvinylidene difluoride (PVDF) membranes. After blocking the membranes with bovine serum albumin and incubating them with primary (1:1000) and secondary antibodies, we exposed them to ECL Plus (GE Healthcare) and visualized the protein bands with the ChemiDoc imaging system (Bio-Rad).

### Statistical Analysis

**Figure 2:**
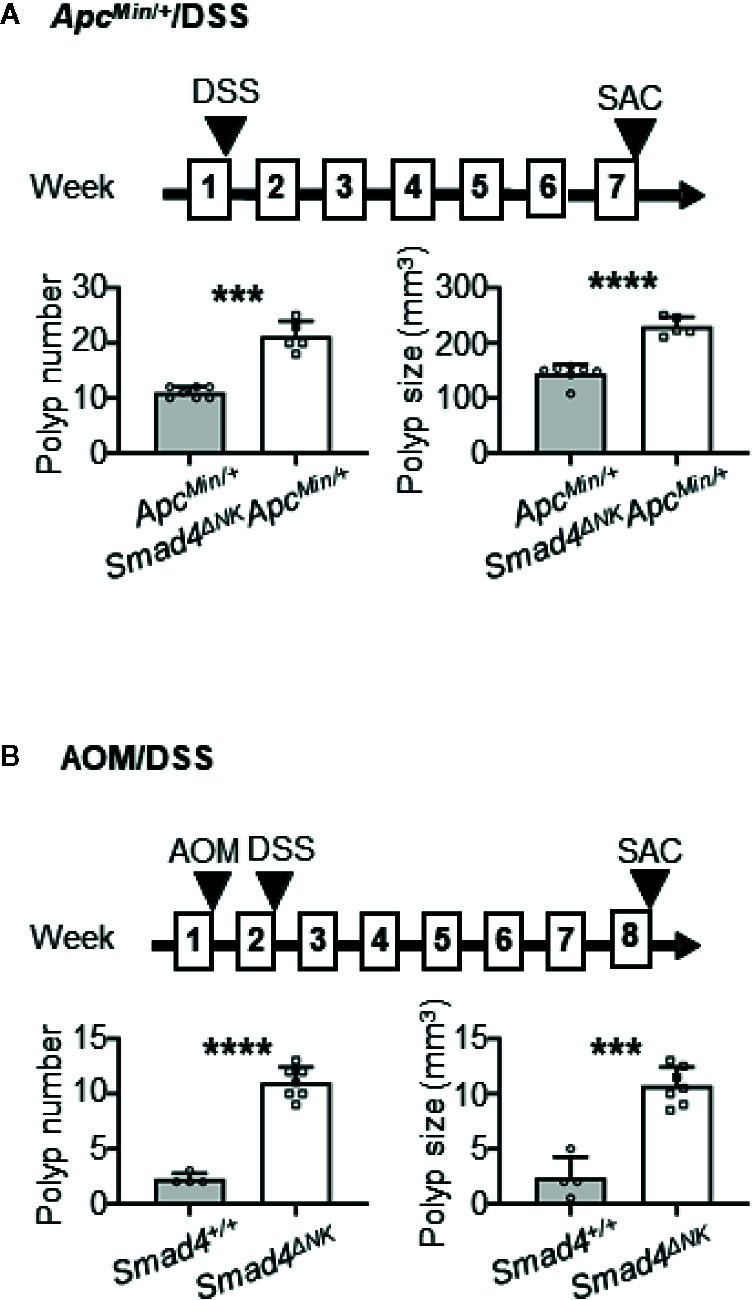
Deletion of Smad4 in NK cells promoted colon adenoma in the *Apc^Min/+^*/dextran sulfate sodium (DSS) mouse model, and promoted adenocarcinoma in the azoxymethane (AOM)/DSS model. **(A)** Evaluation of adenoma numbers and sizes in *Apc^Min/+^* and *Smad4^ΔNK^Apc^Min/+^* mice treated overnight with DSS (n=5–7). **(B)** Evaluation of adenocarcinoma numbers and sizes in *Smad4^+/+^* and *Smad4^ΔNK^* mice treated with AOM and then overnight with DSS (n=4–7). SAC: mouse euthanized. The circles and squares on the bar graphs denote data from individual mice. ***p < 0.001; ****p < 0.0001.

**Figure 3:**
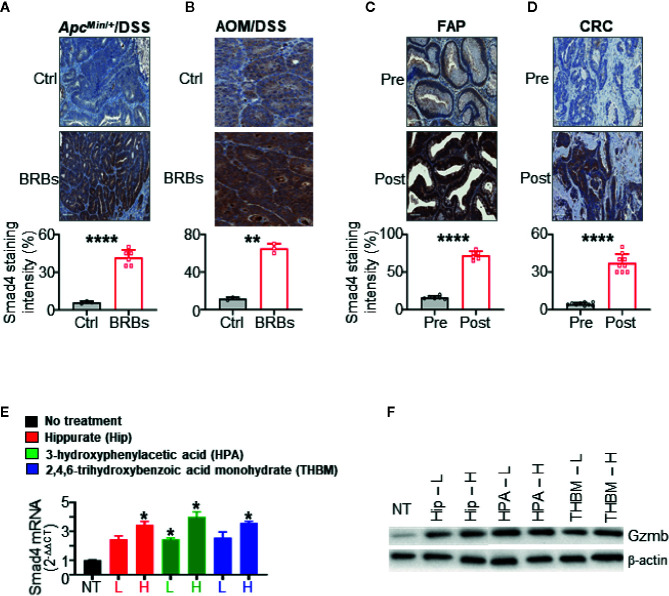
Black raspberries (BRBs) increased Smad4 expression in colorectal cancer (CRC) mouse models and primary colon specimens. Representative immunohistochemistry (IHC) images and quantitative staining of Smad4 in colon sections from **(A)** *Apc^Min^* ^/+^ mice treated overnight with dextran sulfate sodium (DSS) and **(B)** mice treated with azoxymethane (AOM) followed by overnight DSS. The mice were fed control (Ctrl) or BRB diets. Representative IHC images and quantitative staining of Smad4 in sections of **(C)** colon adenoma from FAP patients (n=6) and **(D)** colon adenocarcinoma from CRC patients (n=9). The circles and squares on the bar graphs denote data from individual specimens. Benzoate metabolites from consumed BRBs upregulate Smad4 mRNA expression **(E)**, Gzmb protein expression **(F)** in primary human NK cells (n=5 human donors). NT, no treatment, L, low, 100 nM, H, high, 1 µM. *P < 0.05; **P < 0.01; ****P < 0.0001.

We conducted normality test to show the validity of using t-tests for our analysis. In order to have enough sample size to run the normality test, we standardized and pulled the same measurements from different experiments together (e.g., Smad4 intensity for **Figures 1B–D** and **Figures 3A–D**; Polyp number and Polyp size for **Figures 2A, B**) to test the normality assumption. Based on Shapiro-Wilk normality test, we obtained *p*-value = 0.156 for Smad4 intensity, *p*-value = 0.057 for Polyp number, and *p*-value = 0.482 for Polyp size, which suggest the normality assumption hold for these three measurements given 0.05 significance level. Accordingly, GraphPad Prism 8 (San Diego, CA) was used to perform unpaired two-tailed Student’s *t*-tests to determine changes between two groups. A *P*-value less than 0.05 was considered statistically significant.

## Results

### Smad4 Expression Is Dampened in Circulating NK Cells and Colonic Epithelium in CRC Patients

We previously showed that levels of infiltrating NK cells were lower in adenomas from FAP patients than in adenocarcinomas from CRC patients (40). In the current study, we first measured expression levels of Smad4 in CRC. NK cells were isolated from peripheral blood mononuclear cells (PBMC) of healthy donors (n=10) and untreated metastatic CRC patients (n=24). Our data showed that Smad4 mRNA expression (Smad4/Gapdh) in NK cells from the CRC patients was significantly lower than that in the healthy donors (**Figure 1A**). This suggests that the anti-tumor effects of NK cells might be impaired in CRC patients because of lower Smad4 levels. Because the role of Smad4 in NK cells in human CRC has been unclear, our results support the hypothesis that Smad4 might control the functions of NK cells in CRC carcinogenesis. We also assessed levels of Smad4 protein in colorectal tissues from FAP and CRC patients, using IHC (**Figure 1B**). Paraffin-embedded tissues of adenomas from FAP patients (n=6) and adenocarcinomas from CRC patients (n=9) were stained for Smad4. Staining was strong in colonic crypts from FAP patients, and was dramatically lower in adenocarcinomas than in adenomas (**Figure 1B**). As loss or weak expression of Smad4 associates with poor survival in CRC patients (14, 15), our results support the hypothesis that loss of Smad4 contributes to the progression of CRC (16).

### Smad4 Expression Decreases in CRC Mouse Models

We then investigated Smad4 expression in CRC mouse models. We found lower Smad4 expression with disease progression in *Apc^Min/+^* mice treated overnight with 5% DSS (**Figure 1C**). Smad4 expression was also drastically lower with disease progression in wild-type mice treated with a single dose of AOM (15 mg/kg body weight) followed by overnight DSS (**Figure 1D**). As shown in **Figure 1**, staining for Smad4 was evident largely in crypts in both mouse (**Figures 1C, D**) and human colons (**Figure 1B**). Accordingly, these mouse CRC models induced by mild inflammation reasonably recapitulate the progression of adenoma to adenocarcinoma in sporadic human CRC that is accompanied by loss of Smad4.

### Deletion of Smad4 Specifically in NK Cells Promotes the Development of Colon Adenomas in the *Apc* ^Min/+^/DSS Mouse Model and of Adenocarcinomas in the AOM/DSS Model

To determine if NK cells are important guardians against CRC progression, we previously used anti-NK1.1 antibody to deplete NK cells (Note: NK1.1 is another marker for murine NK cells) and the antibody-treated animals developed more and larger lesions in the colon (40). In the current study, we investigated the contribution of Smad4 in NK cells to CRC progression. Mice with iCre under the control of the NK-specific NK^p46^ promoter (NK^p46-iCre^ mice) were crossed with *Smad4* ^fl/fl^ mice to generate NK^p46iCre/+^ *Smad4* ^fl/fl^ mice in which Smad4 is deleted only in NK cells (*Smad4* ^ΔNK^ mice). Western blotting demonstrated that NK cells isolated from those mice did not express Smad4 (19). *Apc* ^Min/+^ mice were then crossed to *Smad4* ^ΔNK^ mice to generate *Apc* ^Min/+^ mice carrying the Smad4 deletion specifically in NK cells (*Smad4* ^ΔNK^ *Apc* ^Min/+^ mice). The *Smad4* ^ΔNK^ *Apc* ^Min/+^ mice and the control *Apc* ^Min/+^ (with wild-type Smad4) mice were exposed overnight to 5% DSS in drinking water, and sacrificed 6 weeks later (**Figure 2A**). Our data showed that the *Smad4* ^ΔNK^ *Apc* ^Min/+^ mice developed significantly more and larger adenomas in the colon (**Figure 2A**). In another model, *Smad4* ^ΔNK^ and wild-type mice were treated with one dose of AOM and then 5% DSS in their drinking water overnight (**Figure 2B**). Those mice were sacrificed 5 weeks later. The *Smad4* ^ΔNK^ mice developed significantly more and larger adenocarcinomas in the colon (**Figure 2B**). Our data suggest that Smad4 in NK cells could play crucial roles in guarding against CRC progression. More importantly, these data support our findings (**Figure 1A**) that Smad4 mRNA expression in NK cells from CRC patients is significantly lower than in healthy individuals, which might lead to CRC progression (**Figure 1A**).

### BRBs Increase Smad4 Expression in CRC Mouse Models and Colonic Epithelium of FAP and CRC Patients

We previously found that BRBs suppress the progression of colonic microadenoma to adenoma in the *Apc* ^Min/+^/DSS model and of colonic adenoma to adenocarcinoma in the AOM/DSS model (40). Also, administration of dietary BRBs correlated with increased levels of tissue-infiltrating NK cells in both models (40). BRB-fed *Apc* ^Min/+^/DSS mice developed fewer and smaller lesions, mostly low-grade dysplasia (40), suggesting that BRBs suppress the progression of microadenoma to adenoma in this model. We stained colon specimens from both *Apc* ^Min/+^/DSS and AOM/DSS mice fed BRBs, as described previously (40). In both models, BRB administration significantly increased Smad4 staining in colon sections than control diet-fed mice (**Figures 3A, B**). Quantitative results indicated significantly increased Smad4 protein expression in epithelium of colon sections from BRB-fed mice (**Figures 3A, B**).

We previously completed two clinical trials that demonstrated beneficial effects of BRBs for FAP and CRC patients (41, 44), as BRBs increased the number and cytotoxicity of tumor-infiltrating NK cells (40). In the current study, we examined Smad4 expression in specimens from those two trials. In colorectal sections from patients who had consumed BRBs, we observed increased Smad4 staining in the epithelium (**Figures 3C, D**). Quantitative results indicated a significantly higher level of Smad4 staining in colorectal tissue from the post-BRB group compared with the pre-BRB group (**Figures 3C, D**). Taken together, these findings show that BRBs enhance Smad4 expression in colonic epithelium in both mouse CRC models and patients with CRC or FAP.

### Benzoate Metabolites From BRBs Upregulate Smad4 Expression in Primary Human NK Cells

We studied three metabolites—hippurate, 3-hydroxyphenylacetic acid, and 2,4,6-trihydroxybenzoic acid monohydrate—whose levels increase significantly (>10-fold) after BRB intake and/or are known to result from gut microbial metabolism (15, 27, 45, 46). To determine if these metabolites could regulate Smad4 mRNA expression in primary human NK cells (n=5 human donors), we used concentrations 100 nM and 1 μM, as benzoate metabolites are detected at 0.01–1 μM in plasma from humans who consume berries (47, 48). Each of the three metabolites significantly increased the expression of Smad4 (**Figure 3E**) and Gzmb (**Figure 3F**). These data suggest that hippurate, 3-hydroxyphenylacetic acid, and 2,4,6-trihydroxybenzoic acid monohydrate might modulate Smad4 signaling in NK cells to regulate maturation, homeostasis, and anti-tumor immunity. In addition to the awareness that nutrients or phytochemicals might modulate NK cell function (21), our studies provide new evidence that gut bacterial metabolites could modulate NK cells through Smad4 signaling to fight CRC.

## Discussions

The *Apc^Min/+^* mouse model is an imperfect approximation of CRC because its tumors develop in the intestine rather than the colon. We therefore used abbreviated DSS treatment to establish a CRC model on the *Apc^Min/+^ background*. To avoid excessive inflammation, we administered DSS only overnight to slightly irritate the colon. Two and 4 days after the DSS treatment ended, we saw only minor colonic mucosal epithelial injury and focal erosion/ulceration (40). The incidence of colon tumors in both the *Apc* ^Min/+^ mice and AOM-treated mice on the C57/B6 background was 100% (40). These models therefore reasonably mimic the mild inflammation that promotes sporadic human CRC, and represent unique tools for studying CRC.

Our group previously reported that Smad4 regulates murine NK cell homeostasis and maturation and anti-tumor Immunity (19). Using a metastatic melanoma model (in which B16F10 cells were injected i.v. with a melanoma cell line that could kill NK cells and metastasize to the lungs), we showed that selective deletion of Smad4 from murine NK cells dramatically reduced the rejection of tumor cells, augmenting tumor cell metastasis and impeding NK cell maturation and homeostasis (19). This associated with downregulation of granzyme B (Gzmb), Kit, and Prdm1 in Smad4-deficient NK cells. We also determined how Smad4 promotes Gzmb expression: Gzmb was identified as a direct target of a transcriptional complex formed by Smad4 and JunB. A JunB-binding site in the proximal Gzmb promoter— which was distinct from the binding site for Smad4—was required for transcriptional activation by the Smad4-JunB complex. Accordingly, our study identified the pathways and mechanisms that govern Smad4’s innate immune responses to cancer as well as its role in NK cell development. Whether NK cells use this same pathway to regulate their functions during CRC progression warrants further investigation.

Dietary constituents are metabolized in the gut and transformed into bioactive compounds by gut microbes (45). We used a metabolomic approach to identify potential bioactive metabolites of BRBs in urine of CRC patients (44). BRB intervention significantly increased levels of several benzoate metabolites (44), which others have identified as bacterial metabolites of polyphenols in BRBs (49). In addition, BRBs modulated levels of metabolites that associate with pathways such as amino acid metabolism and the TCA cycle (44). Collectively, our data suggest that BRBs are both a target of gut metabolism and may also influence metabolism by gut bacteria.

Previous research found that Smad4 loss is associated with poor survival, resistance to chemotherapy and increased metastasis (50–53). However, the mechanisms by which loss of Smad4 contributes to tumor progression and metastasis are poorly understood and they are likely complex, given the tissue-specific roles of Smad4 signaling and the multiple processes that it regulates. One possibility is that the loss of Smad4 signaling in tumor epithelial cells results in tumor-promoting activation of the tumor microenvironment through mechanisms of intercellular crosstalk. To determine the specific effects of BRBs on Smad4 signaling in both epithelium and NK cells, single cell RNAseq experiments on both cell types collected before and after BRB treatment are required to accurately answer this question.

## Conclusions

Our current study suggests that the tumor suppressive actions of Smad4 in both colonic epithelium and NK cells could interfere with the transformation of benign to malignant stages of CRC. BRB components and their benzoate metabolites could upregulate Smad4 to suppress preneoplastic colonic epithelium and enhance NK cell function to delay CRC progression. Accordingly, our findings could provide a foundation for developing NK-focused therapeutics for CRC, with the bonus of targeting colonic epithelium at the same time. Most encouragingly, we identified several benzoate metabolites from an edible fruit that upregulates Smad4 in colonic epithelium and NK cells. Importantly, these metabolites are also produced during intestinal metabolism of many commonly consumed plant-based foods such as fruits and vegetables and whole grains. As such, the encouragement of consumption of locally produced plant-based foods could be an affordable approach for delaying the progression of CRC in humans around the world.

## Data Availability Statement

The raw data supporting the conclusions of this article will be made available by the authors, without undue reservation.

## Ethics Statement

The studies involving human participants were reviewed and approved by The institutional review boards of The Ohio State University, University of Texas, San Antonio, and Medical College of Wisconsin. The patients/participants provided their written informed consent to participate in this study. The animal study was reviewed and approved by The Medical College of Wisconsin Animal Care and Use Committee.

## Author Contributions

JY and L-SW designed the research; Y-WH, C-WL, PP, TS, CE, Y-YM, H-ZW, MA, ST, KO, and MY conducted the research and analyzed the data; JX, HC, CS, MD, and WB discussed the concept; JY, and L-SW wrote the paper. All authors contributed to the article and approved the submitted version.

## Funding

This work was supported by NIH grants CA148818 and USDA/NIFA 2020-67017-30843 (to L-SW) and CA185301, AI129582, and NS106170 (to JY).

## Conflict of Interest

The authors declare that the research was conducted in the absence of any commercial or financial relationships that could be construed as a potential conflict of interest.
