# Supplementary material for: Black Raspberries Suppress Colorectal Cancer by Enhancing Smad4 Expression in Colonic Epithelium and Natural Killer Cells
Source: Front Immunol. 2020 Dec 14;11:570683. doi: 10.3389/fimmu.2020.570683 (PMC7793748; doi:10.3389/fimmu.2020.570683)
Supplement: Supplementary file 1 [file Table_1.docx]

**Supplementary Table 1. Clinical information for human samples used in Figure 1A and 1B.**

| **Figure 1A** | **Subjects** | **Histology** | **Gender** | **Age** |
| --- | --- | --- | --- | --- |
| 1 | Healthy subject | N/A (Not Available) | F | 85 |
| 2 | Healthy subject | N/A | M | 76 |
| 3 | Healthy subject | N/A | M | 46 |
| 4 | Healthy subject | N/A | M | 57 |
| 5 | Healthy subject | N/A | M | 53 |
| 6 | Healthy subject | N/A | F | 30 |
| 7 | Healthy subject | N/A | F | 41 |
| 8 | Healthy subject | N/A | F | 45 |
| 9 | Healthy subject | N/A | F | 35 |
| 10 | Healthy subject | N/A | F | 50 |
| 1 | Colorectal cancer | Adenocarcinoma | M | 72 |
| 2 | Colorectal cancer | Adenocarcinoma | F | 67 |
| 3 | Colorectal cancer | Adenocarcinoma | M | 49 |
| 4 | Colorectal cancer | Adenocarcinoma | F | 76 |
| 5 | Colorectal cancer | Adenocarcinoma | F | 64 |
| 6 | Colorectal cancer | Adenocarcinoma | M | 62 |
| 7 | Colorectal cancer | Adenocarcinoma | M | 26 |
| 8 | Colorectal cancer | Adenocarcinoma | F | 71 |
| 9 | Colorectal cancer | Adenocarcinoma | F | 79 |
| 10 | Colorectal cancer | Adenocarcinoma | M | 45 |
| 11 | Colorectal cancer | Adenocarcinoma | M | 55 |
| 12 | Colorectal cancer | Adenocarcinoma | F | 37 |
| 13 | Colorectal cancer | Adenocarcinoma | F | 63 |
| 14 | Colorectal cancer | Adenocarcinoma | M | 47 |
| 15 | Colorectal cancer | Adenocarcinoma | F | 44 |
| 16 | Colorectal cancer | Adenocarcinoma | F | 55 |
| 17 | Colorectal cancer | Adenocarcinoma | M | 75 |
| 18 | Colorectal cancer | Adenocarcinoma | M | 37 |
| 19 | Colorectal cancer | Adenocarcinoma | M | 60 |
| 20 | Colorectal cancer | Adenocarcinoma | F | 75 |
| 21 | Colorectal cancer | Adenocarcinoma | F | 57 |
| 22 | Colorectal cancer | Adenocarcinoma | M | 60 |
| 23 | Colorectal cancer | Adenocarcinoma | F | 85 |
| 24 | Colorectal cancer | Adenocarcinoma | F | 62 |
|  |  |  |  |  |
| **Figure 1B** | **Subjects** | **Histology** | **Age** | **Gender** |
| 1 | Colorectal cancer | Adenocarcinoma | F | 65 |
| 2 | Colorectal cancer | Adenocarcinoma | F | 74 |
| 3 | Colorectal cancer | Adenocarcinoma | F | 68 |
| 4 | Colorectal cancer | Adenocarcinoma | M | 55 |
| 5 | Colorectal cancer | Adenocarcinoma | M | 63 |
| 6 | Colorectal cancer | Adenocarcinoma | M | 69 |
| 7 | Colorectal cancer | Adenocarcinoma | F | 51 |
| 8 | Colorectal cancer | Adenocarcinoma | M | 54 |
| 9 | Colorectal cancer | Adenocarcinoma | F | 53 |
| 1 | Familial adenomatous polyposis | Tubular adenoma | M | 65 |
| 2 | Familial adenomatous polyposis | Tubular adenoma | F | 68 |
| 3 | Familial adenomatous polyposis | Tubular adenoma | F | 70 |
| 4 | Familial adenomatous polyposis | Tubular adenoma | M | 56 |
| 5 | Familial adenomatous polyposis | Tubular adenoma | M | 51 |
| 6 | Familial adenomatous polyposis | Tubular adenoma | F | 52 |
